# CASP4 can be a diagnostic biomarker and correlated with immune infiltrates in gliomas

**DOI:** 10.3389/fonc.2022.1025065

**Published:** 2023-01-11

**Authors:** Guopeng Tian, Qiao Li, Liang Niu, Yusong Luo, Hongyu Wang, Wei Kang, Xiang Fang, Shengwei Bai, Guoqiang Yuan, Yawen Pan

**Affiliations:** ^1^ Department of Neurosurgery, Lanzhou University Second Hospital, Lanzhou, China; ^2^ Laboratory of Neurology of Gansu Province, Lanzhou University Second Hospital, Lanzhou, China

**Keywords:** glioma, pyroptosis, CASP4, biomarker, immune infiltration

## Abstract

**Background:**

Gliomas are the most common and invasive malignant tumors that originate in the central nervous system. Currently, the primary treatment modality for gliomas is maximum surgical resection, supplemented by radiotherapy and chemotherapy. However, the long-term survival rate has not signifificantly increased. Pyroptosis is a new form of programmed lytic death that has been recently discovered. Caspase 4 (CASP4) plays a key role in pyroptosis. Many studies have shown that pyroptosis is not only related to inflflammation but is also closely related to the occurrence and development of most tumors. This study aimed to prove that CASP4 has a key role in the mechanism of gliomas.

**Methods:**

We used expression data from The Cancer Genome Atlas and the Chinese Glioma Genome Atlas to explore the relationship between CASP4 expression and glioma prognosis. The differential expression of CASP4 in gliomas and normal tissues was fifirst tested, and then the connection between CASP4 and tumor prognosis was explored. The relationship between CASP4 expression and immune cell infifiltration was also investigated. Finally, the possible pathways were analyzed using Gene Ontology, Kyoto Encyclopedia of Genes and Genomes, and Gene Set Enrichment Analysis.

**Results:**

CASP4 was highly expressed and associated with a signifificantly lower survival rate in patients with glioma. It could also inflfluence immune cell infifiltration by releasing cytokines.

**Conclusion:**

CASP4 can be a diagnostic biomarker and is a promising therapeutic target for gliomas.

## Introduction

Gliomas, which are derived from neuroglial stem or progenitor cells, are the most common type of malignant tumor of the central nervous system (CNS). Gliomas account for almost 30% of all primary brain tumors and 80% of all malignant ones (1). Gliomas are categorized into World Health Organization (WHO) grades 1–4 based on their different degrees of malignancy (2). As a refractory tumor, the standard treatment for gliomas includes maximum safe resection combined with concurrent radiotherapy and temozolomide chemotherapy followed by temozolomide. However, despite surgical resection, chemotherapy, and radiotherapy, the median survival time after diagnosis is only 14–16 months (3, 4), and the 5-year survival rate is only 6.8% (5). Inflammation is a basic feature of glioblastoma (GBM) biology (6), but its role in gliomas has not been elucidated. The main manifestation of this inflammation is the lack of T cell infiltration (7), but a large number of tumor-associated macrophages and tumor-associated lymphocytes are infiltrated and activated (8). Inflammation in gliomas is associated with poor prognosis and reduced tumor response to treatment (9). Tumor-associated macrophages can affect the proliferation of tumor cells, which is closely related to their polarization or differentiation (10).

Microglia, the innate immune cells, reside in the CNS and constitute a group of macrophages, also known as brain intrinsic cells (11). Increasing evidence shows that microglia are no longer simply inflammatory cells (12). Glioma tissues contain nearly 30% microglia (13). Under the induction of glioma cells, microglia differentiate into a certain phenotype and gather at the edge of the tumor, promoting the invasion and growth of glioma. In addition, the density of microglia is positively correlated with the benign, malignant, and invasive nature of the tumor (12, 14). Microglia also stimulate tumor-associated angiogenesis and tumor cell migration (15). Pyroptosis is a newly discovered method of programmed cell lytic death that mainly plays a role in resisting the invasion of external pathogens and sensing endogenous danger signals (16). The latest definition of pyroptosis by the Nomenclature Committee Cell Death in 2018 is regulated cell death caused by the formation of plasma membrane pores mediated by gasdermin (GSDM) family proteins (GSDMA–GSDME), often but not always due to the activation of inflammatory caspases (17).

Cysteine aspartate proteases (caspases, CASP) are a group of highly conserved intracellular proteases (18). They are similar in structure and have active sites that contain cysteine residues, which can specifically break peptide bonds after aspartic acid residues. As a key molecule in the non-classical pathway of cell death, CASP4 is the core component of atypical inflammatory bodies (19) and plays an important role in pyroptosis. Intracellular lipopolysaccharide from gram-negative bacteria is a molecular model associated with bacterial pathogens that promotes the activation of CASP4 (20, 21). CASP4 acts directly as a receptor for cytoplasmic bacterial lipopolysaccharide. CASP4 is activated by lipopolysaccharide binding, which triggers pyroptosis (22). CASP4 also depends on the activation of NLRP3 and CASP 1 through their effect on IL1β/18 maturation indirectly (23, 24). Increasing evidence suggests that pyroptosis is not only related to inflammation but is also closely associated with the development and occurrence of tumors. The role of pyroptosis in neoplastic diseases appears to be a double-edged sword. On the one hand, pyroptosis can quickly lead to tumor regression. However, it can also promote tumor development. Therefore, tumor cells may inhibit or stimulate pyroptosis to support its progression, depending on the microenvironment.

This study aimed to explore the mechanism of CASP4 in gliomas. Towards this goal, bioinformatic methods were used to determine the potential role of CASP4 in gliomas. Data were collected from The Cancer Genome Atlas (TCGA) and Chinese Glioma Genome Atlas (CGGA).

## Materials and methods

### Data acquisition

We collected tumors from 29 patients with gliomas, including low-grade glioma (LGG) and GBM, and normal tissues from 14 patients who underwent surgical treatment at the Department of Neurosurgery, Lanzhou University Second Hospital between May 2020 and January 2022. None of the 43 patients received any other treatment before surgery.

This study was approved by the Ethics Committee of Lanzhou University Second Hospital and was conducted according to the tenets of the Helsinki Declaration. Informed consent was obtained from all the patients.

### CASP4 expression in TCGA and CGGA database

Data from TCGA database (https://genome-cancer.ucsc.edu/) and CGGA database (http://www.cgga.org.cn) were collected to evaluate CASP4 mRNA expression. HTSeq-FPKM data were used for subsequent analyses. The samples were divided into the low-expression group and the high-expression group according to the median CASP4 expression to analyze the association between overall survival (OS) and CASP4 expression.

### Quantitative real-time polymerase chain reaction

CASP4 expression was detected using quantitative real-time polymerase chain reaction. Total RNA was extracted from glioma tissues using the Trizol kit (Invitrogen) according to the manufacturer’s instructions. A reverse transcription kit (Takara) was used for cDNA synthesis. GAPDH was used as an internal control. The primer sequences were as follows: CASP4 forward primer: 5′-AAGAGAAGCAACGTATGGCAGGAC-3′ and CASP4: reverse primer, 5′-GGACAAAGCTTGAGGGCATCTGTA-3′ as well as GAPDH forward primer: 5′-GGACCTGACCTGCCGTCTAG-3′ and GAPDH reverse primer: 5′-TAGCCCAGGAGGATGCCCTTGAG-3′.

### Western blot

Radioimmunoprecipitation assay lysis buffer was used for tissue protein extraction and quantification. Proteins were separated on 12% sodium dodecyl sulfate–polyacrylamide gel and then transferred onto polyvinylidene fluoride membranes. The polyvinylidene fluoride membranes were blocked with a blocking solution (P0252, Beyotime) at room temperature for 1.5 h and then incubated overnight with primary antibodies at 4°C. On the next day, the membranes were incubated with secondary antibodies at room temperature for 2 h, and immunoreactive signals were detected by western blotting. The primary antibodies used were rabbit anti-human CASP4 monoclonal antibody (30 kDa; 1.5:1,000; PA5-20109, Thermo Fisher) and mouse anti-human tubulin antibody (55 kDa; 1:1,000; ab44928, Abcam).

### Immunohistochemistry

Glioma tissue were fixed in formalin, embedded in paraffin, and cut into 4-μm-thick sections. Slides were prepared by dewaxing and hydration. Antigen retrieval was accomplished by boiling the sections for 15 min in sodium citrate buffer at 95°C, dissolving with endogenous peroxidase in 3% hydrogen peroxide, and blocking with 1% bovine serum albumin. Primary antibodies were incubated overnight at 4°C with rabbit anti-human CASP4 monoclonal antibody (PA5-20109, Thermo Fisher). Following the treatment, the slides were stained with secondary goat anti-rabbit IgG antibodies [UltraSensitiveTM SP (mouse/rabbit) IHC Kit, KIT-9710, MXB]. We used 3,3′-diaminobenzidine for staining and Harris hematoxylin for restraining. Finally, the slides were dehydrated and covered with neutral resin. The samples were scanned using a Leica microscope. The evaluation focused on cytoplasmic staining. The integrated optical density value of the dyed region in the immunohistochemistry section was evaluated using the Image-Pro Plus software (version 6.0, Media Cybernetics Inc., Rockville, MD, USA). The average optical density (integrated optical density/area) was used as an indicator of stain intensity in the CASP4 stain. Three fields were randomly selected from each tissue section (magnification, ×200) for capture. Statistical analysis was performed using the average optical density values.

### UALCAN and GEPIA2 analyses

The correlation between CASP4 expression and the clinicopathological characteristics of glioma patients (tumor grade, race, age, and histological type) and the potential prognostic value of CASP4 expression in gliomas of different grades (25) were analyzed using UALCAN (ualcan.path.uab.edu) (26) and GEPIA2 (gepia2.cancer-pku.cn) (27). These are publicly accessible databases for an in-depth analysis of the gene expression data from TCGA.

### Immune infiltration and functional enrichment analyses

A previous study identified 24 immune cells using genetic markers (28). The three HTSeq-FPKM format RNA-seq data based on TCGA-GBMLGG (gliomas) (https://portal.gdc.cancer.gov/) were parallel log2-converted and used to determine the degree of tumor immune infiltration. Using the GSVA R package (version 1.34.0) (29), we identified the above-mentioned data using single-sample GSEA. The correlation between CASP4 and the 24 immune cell types was analyzed using Spearman’s correlation test. Graphs and figures were created using the ggplot2 package in R.

### Functional enrichment analysis

Glioma-related genes were downloaded, and CASP4-related prognostic genes were screened using TCGA database. STRING (https://string-db.org/) was used to build a protein–protein interaction (PPI) network of CASP4 co-expressed genes, and the data were imported into Cytoscape for further analysis. The “clusterProfiler” R package (version 3.14.3) (30) was used for Gene Ontology (GO) and Kyoto Encyclopedia of Genes and Genomes (KEGG) enrichment analyses to identify possible biological functions and signaling pathways. The GO analysis included biological processes (BPs), cell composition, and molecular function (*P* < 0.05, statistically significant). Gene set enrichment analysis (GSEA) was used to determine significant and consistent differences between two biological states of a set of genes. To identify CASP4-related signaling pathways, data downloaded from TCGA database were used to divide tumor samples into low- and high-CASP4-expression groups, with normalized enrichment score >1.5 and nominal *P* < 0.05 (31) considered in the analysis. C2.all.v7.2.symbols.gmt was used to run the GSEA.

### Statistical analysis

Differences in CASP4 expression between normal tissues and tumor tissues were analyzed using Wilcoxon signed-rank tests and one-way analysis of variance. The association between CASP4 expression and clinicopathological features was analyzed using the Kruskal–Wallis test, Dunn’s test, and Wilcoxon rank-sum test. Diagnostic accuracy was evaluated according to the area under the curve calculated using the receiver operating characteristic (ROC) curve analysis. Moreover, the Kaplan–Meier method and Cox regression analysis were used to evaluate the prognostic significance of CASP4 expression. Independent prognostic factors for gliomas were identified using the R package (version 3.6.3; https://www.r-project.org/). Univariate and multivariate Cox proportional hazard regression analyses were conducted to exclude CASP4 and eight clinico-demographic characteristics [age, isocitrate dehydrogenase (IDH) status, 1p/19q codeletion (32), histological type, sex, grade, race, and primary therapy outcome] from OS analysis. A nomogram was created using the “rms” (version 6.2-0) and “survival ROC” (version 3.2-10) packages in R, and the area under the curve values of each predictor for OS were calculated. In addition, a calibration curve was used to evaluate the performance of the nomogram. All statistical analyses were performed using SPSS for Windows (version 18.0; IBM SPSS Statistics, IBM Corporation, Armonk, NY, USA). Statistical significance was set at *P <*0.05.

## Results

CASP4 expression influenced several factors in gliomas, including clinicopathological variables, tumor microenvironment, immune cell pathway, tumor immune infiltration, and PPI. These results suggested that CASP4 is closely related to immunity and may act a key role in the diagnosis and grading of gliomas.

### Expression patterns of CASP4 in gliomas

**Figure 1:**
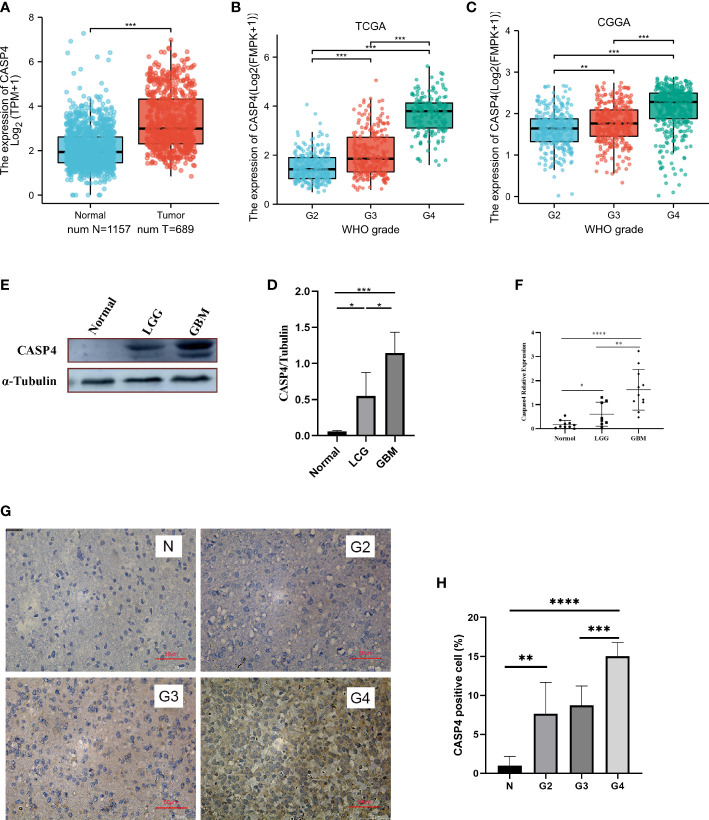
Expression patterns of CASP4 in gliomas. **(A)** Differential expression of CASP4 in different disease states (malignant or benign). The mRNA expression of CASP4 is markedly higher in 689 gliomas in The Cancer Genome Atlas (TCGA) database than in 1,157 normal tissues in the GETx. **(B, C)** Differential expression of CASP4 in different WHO grades. **(B)** TCGA database. **(C)** Chinese Glioma Genome Atlas database. CASP4 expression is significantly different from WHO G2 to G4, and the expression increased as the grade increased. **(D–H)** CASP4 expression at the mRNA and protein levels is verified using **(D,E)** western blot, **(F)** qRT-PCR, and **(G,H)** immunohistochemical staining (10 × 20). The results show that there is no significant difference between them (*P < 0.05, **P < 0.01, ***P < 0.001, ****P < 0.0001). LGG, low-grade glioma; GBM, glioblastoma.

The analysis of the clinical and gene expression data of 689 primary tumors from TCGA, 984 primary tumors from CGGA, and 1,157 normal tissues from GTEx showed that CASP4 expression was higher in tumor tissues than in normal tissues (**Figure 1A**). The glioma grade varied from 2 to 4. For patients in TCGA database, 224 (35.3%), 243 (38.3%), and 168 (26.4%) patients had grade 2, 3, and 4 gliomas, respectively. Meanwhile, for patients in the CCGA database, 279 (28.4%), 322 (32.7%), and 383 (38.9%) patients had grade 2, 3, and 4 gliomas, respectively. CASP4 expression was directly correlated with tumor grade, and the higher the tumor grade, the higher the expression of CASP4 (**Figures 1B, C**). Western blotting (**Figures 1D, E**), quantitative real-time polymerase chain reaction (**Figure 1F**), and immunohistochemistry staining (**Figures 1G, H**) of normal traumatic brain tissues and various grades of gliomas showed consistent findings with those of previous predictions.

### Associations between CASP4 expression and clinicopathologic variables

**Figure 2:**
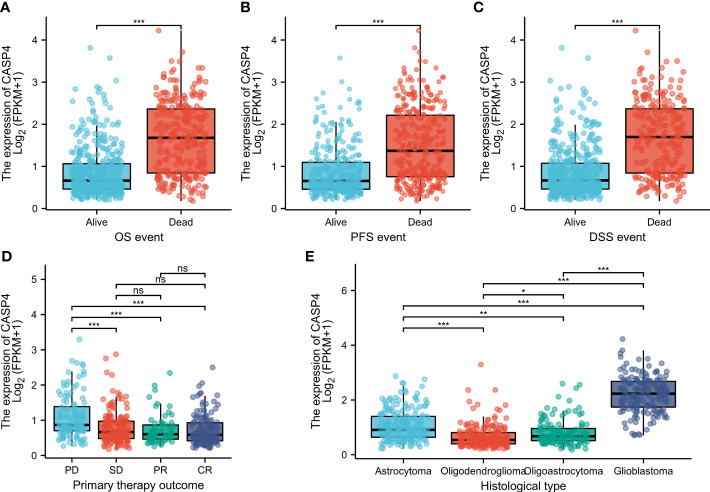
Clinicopathological characteristics associated with CASP4 expression in glioma patients in The Cancer Genome Atlas (TCGA) database. **(A–C)** Association between CASP4 expression and survival. **(A)** Overall survival. **(B)** Progression-free survival. **(C)** Disease-free survival. **(D)** Correlation between CASP4 expression and primary treatment outcome. **(E)** Histological type of glioma correlated with CASP4 expression (*P < 0.05; **P < 0.01; ***P < 0.001; NS, *P* > 0.05). NS is no significance.

The general clinicopathological characteristics of patients in TCGA and CGGA databases are summarized in **Supplementary Table S1**. CASP4 expression was related to the clinicopathological features and prognosis of glioma patients. High CASP4 expression was associated with age, 1p/19q gene deletion, and IDH status but not with sex and race. Furthermore, CASP4 expression was significantly correlated with OS (*P* < 0.001), progression-free survival (*P* < 0.001), and disease-specific survival (*P* < 0.001) (Wilcoxon rank-sum test, **Figures 2A, C**). CASP4 expression was also correlated with primary treatment outcome (*P* < 0.001) and histological type (*P* < 0.001) (Kruskal–Wallis rank-sum test, **Figures 2D, E**).

### High CASP4 expression indicates poor prognosis in glioma patients

**Figure 3:**
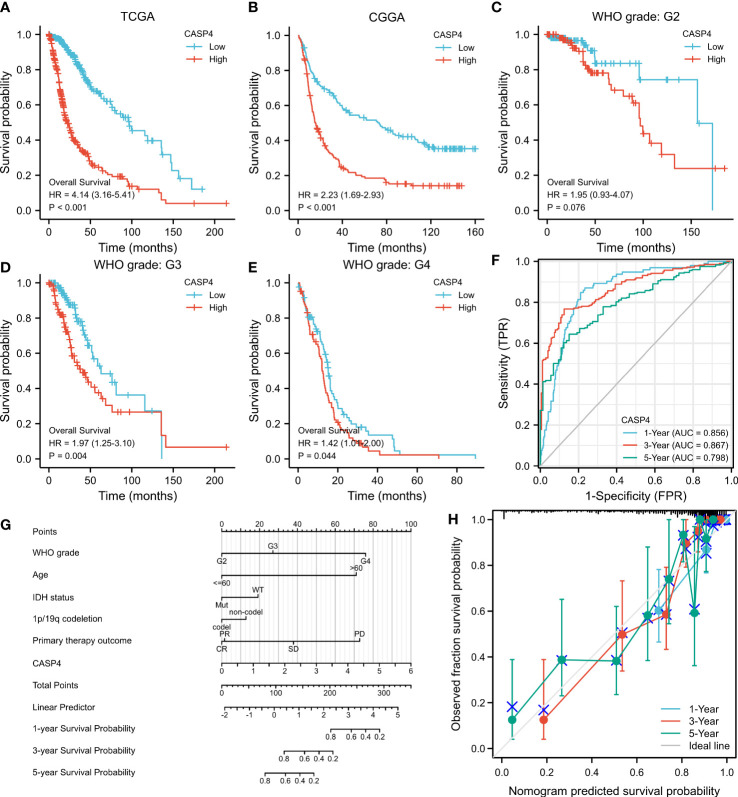
Survival curve, receiver operating characteristic (ROC) curve, and overall survival (OS) nomogram in The Cancer Genome Atlas (TCGA) database and Chinese Glioma Genome Atlas (CGGA) database suggest that high CASP4 expression is related to different disease states (tumor or normal) and overall survival. **(A)** Kaplan–Meier curves of OS in the training set (TCGA). **(B)** OS in the CGGA dataset. **(C–E)** OS by WHO grade in glioma patients in TCGA. **(C)** G2. **(D)** G3. **(E)** G4. **(F)** Time-dependent ROC curves for the 1-, 3-, and 5-year survival in TCGA cohort. **(G)** Nomogram for predicting the probability of 1-, 3-, and 5-year OS of glioma patients. **(H)** Calibration plot of the nomogram for predicting the probability of OS.

A total of 272 samples and 325 samples of gliomas of different grades from TCGA and CGGA, respectively, were included in the analysis. The results showed that the higher the CASP4 expression, the poorer the OS (TCGA: HR = 4.14, 95% CI = 3.16–5.41, Cox *P* < 0.001, **Figure 3A**; CCGA: HR = 2.23, 95% CI = 1.69–2.93, Cox *P* < 0.001, **Figure 3B**). Similarly, a subgroup analysis in TCGA to analyze the prognostic impact of CASP4 in different glioma grades also showed that high CASP4 expression was associated with poorer OS in WHO G3 (HR = 1.97, 95% CI = 0.93–4.07, Cox *P* = 0.004, **Figure 3D**) and WHO G4 (HR = 1.42, 95% CI = 1.01–2.00, Cox *P* = 0.044, **Figure 3E**). Overall, these results suggest that high CASP4 expression is a risk factor of poor prognosis in patients with glioma. In the time-dependent ROC curves to evaluate the usefulness of CASP4 expression for distinguishing between glioma and normal tissues, the area under the curve values for predicting the 1-, 3-, and 5-year survival rates were 0.856, 0.867, and 0.798, respectively (**Figure 3F**). These results suggested that CASP4 expression is a good diagnostic biomarker for glioma. A nomogram was then developed to make the predictive method more intuitive, which could obtain the total points and estimate the 1-, 3-, and 5-year survival rates of glioma patients (**Figure 3G**). Furthermore, the calibration curves (**Figure 3H**) showed that the nomogram satisfactorily predicted the 1-, 3-, and 5- year survival rates.

**Figure 4:**
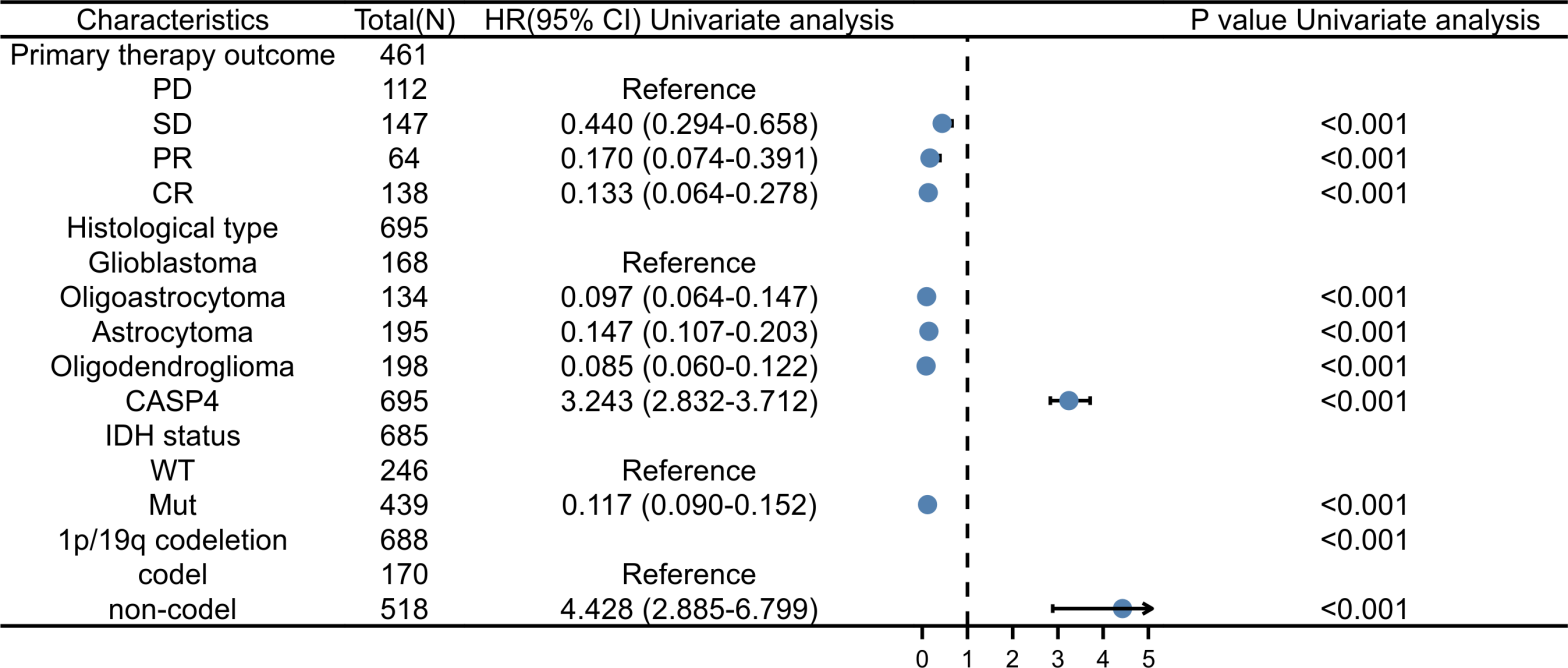
Forest plot for the hazard ratios of CASP4 expression. Association between CASP4 expression and clinical pathological factors based on univariate Cox regression analysis.

As shown in **Supplementary Table S2**, univariate analysis (**Figure 4**) using Cox regression revealed that OS was significantly correlated with IDH status, 1p/19q codeletion, WHO grade, age, histological type, primary treatment outcome, and CASP4 expression level (all *P* < 0.001). Furthermore, the WHO grade, primary treatment outcome, IDH status, and age were independent risk factors of unfavorable OS in the multivariate Cox regression analysis.

### Immune cell infiltration of CASP4

**Figure 5:**
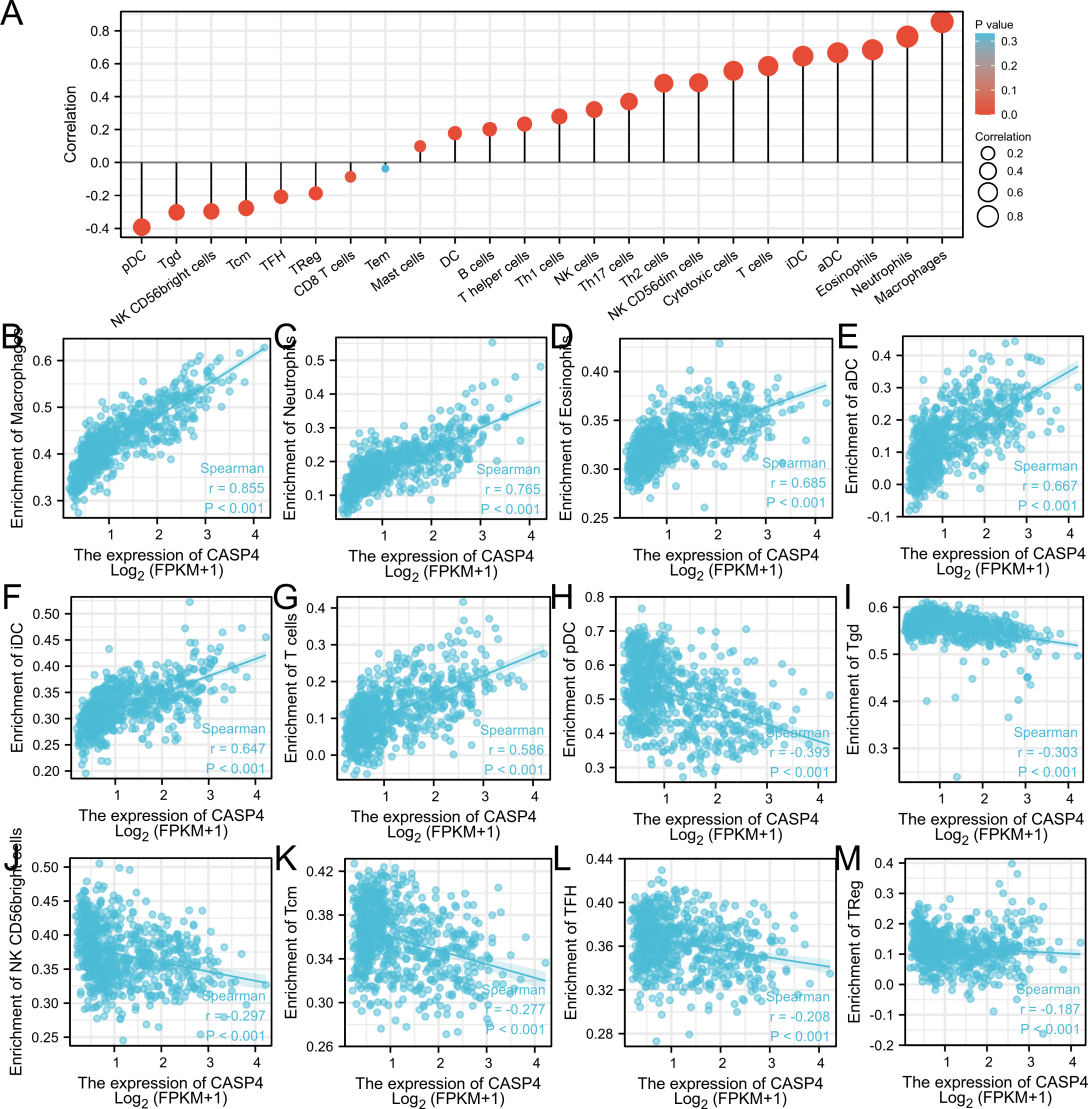
Comparison of immune infiltration in The Cancer Genome Atlas cohort. **(A)** Correlation between infiltrating immune cell types in gliomas and CASP4 expression. **(B–G)** Scatter plot of infiltrating immune cells showing positive correlations with CASP4 expression. **(H–M)** Scatter plot of infiltrating immune cells showing negative correlations with CASP4 expression.

Single-sample GSEA showed that immune-infiltrating cell types were closely related to CASP4 expression (**Figure 5A**). Immune activation-related cells (*e*.*g*., macrophages, neutrophils, eosinophils, antibody–drug conjugates, immature dendritic-like cells, and T cells) were positively correlated with CASP4 expression (**Figures 5B–G**). Meanwhile, plasmacytoid dendritic cells, Tgd, NK CD56 bright cells, Tcm, TFH, TReg, and CD8 T cells were negatively correlated with CASP4 expression (**Figures 5H–M**). These data show that CASP4 strongly promotes immune cell infiltration.

### Enrichment analysis of CASP4-related partners

**Figure 6:**
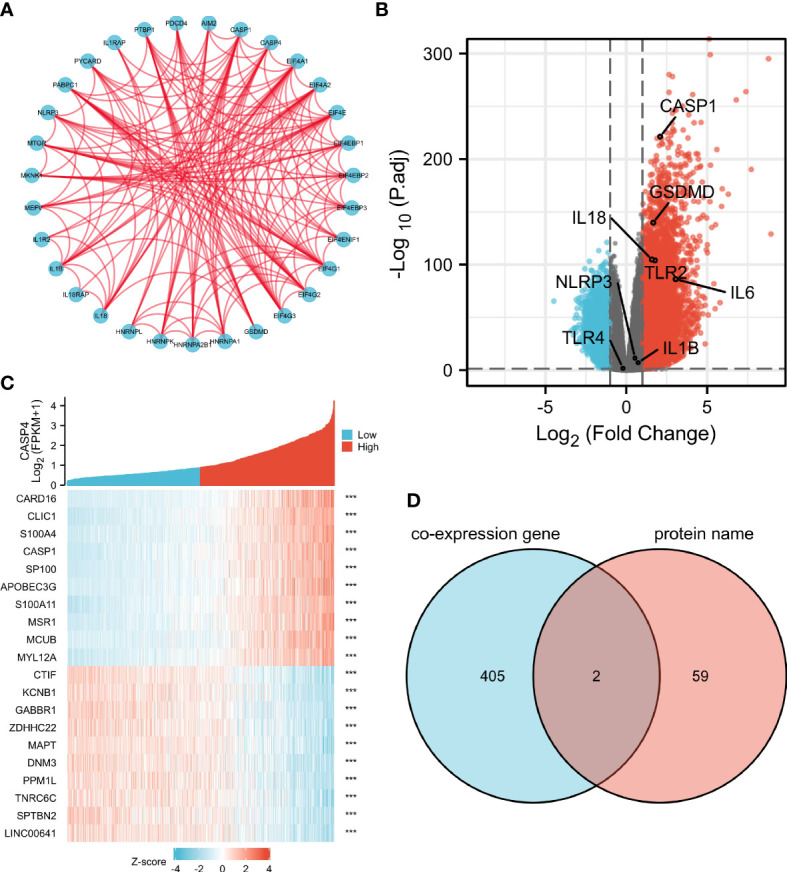
Protein–protein interactions (PPI) and functional analysis of CASP4 and its co-expressed genes in gliomas. **(A)** The PPI analysis of 61 binding proteins is performed using the STRING software. **(B)** Volcano plot of differentially expressed genes between the high- and low-CASP4-expression groups. **(C)** Heat map of the top 10 significantly differentially expressed genes between the high- and low-CASP4-expression groups. **(D)** Venn diagram of the above-mentioned two groups showing two common members, namely, CASP1 and GSDMD.

A total of 61 CASP4-binding proteins were obtained based on the STRING tool. The PPI network created using Cytoscape is shown in **Figure 6A**. We combined all tumor expression data from TCGA with GEPIA2 data and obtained differentially expressed genes that were correlated with CASP4 expression in gliomas, including GSDMD, CASP1, IL-18, and TLR2. A standard |logFC| >0.75 and an adjusted *P*-value <0.001 were used to analyze the differentially expressed genes (DEGs). A total of 407 DEGs between the high- and low-CASP4 groups were identified; among these, 358 and 49 genes were upregulated and downregulated, respectively (**Figure 6B**). The top 10 significantly differentially expressed genes in the high- and low-CASP4-expression groups are displayed in the form of a heat map in **Figure 6C**. Furthermore, we obtained two common members by an intersection analysis of the high and low groups: CASP1 and GSDMD (**Figure 6D**).

**Figure 7:**
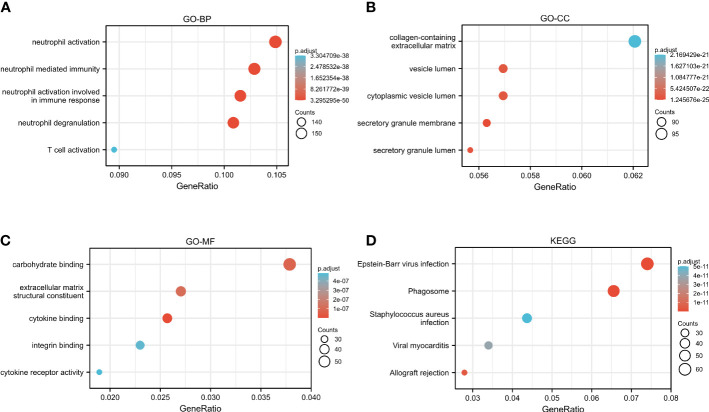
Functional analysis of CASP4 in patients with gliomas. **(A–C)** GO analysis of CASP4 co-expression genes. **(A)** Biological process. **(B)** Cellular component. **(C)** Molecular function. **(D)** KEGG pathway enrichment analysis of CASP4 co-expression genes. GO, Gene Ontology; KEGG, Kyoto Encyclopedia of Genes and Genomes.

The GO and KEGG analyses to identify CASP4 co-expressed genes suggested that CASP4 co-expressed genes were mainly involved in neutrophil activation, neutrophil-mediated immunity, neutrophil activation involved in immune response, neutrophil degranulation, secretory granule lumen, cytoplasmic vesicle lumen, vesicle lumen, cytokine binding, integrin binding, extracellular matrix structural constituent, and carbohydrate binding (**Figures 7A–C**). The KEGG analysis revealed that CASP4-co-expressed genes were enriched in phagosomes, Epstein–Barr virus (EBV) infection, allograft rejection, viral myocarditis, and *Staphylococcus aureus* infection (**Figure 7D**).

### GSEA identification of CASP4-related signaling pathways

**Figure 8:**
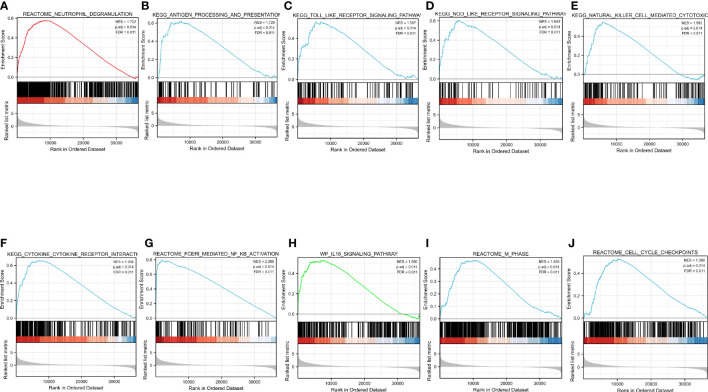
Enrichment pathways from Gene Set Enrichment Analysis. **(A)** Neutrophil degranulation. **(B)** Antigen processing and presentation. **(C)** TOLL-like receptor signaling pathway. **(D)** NOD-like receptor signaling pathway. **(E)** Natural-killer-cell-mediated cytotoxicity. **(F)** Cytokine–cytokine receptor interaction. **(G)** FCERI-mediated NF-kB activation. **(H)** IL18 signaling pathway. **(I)** Cell cycle checkpoints. **(J)** M phase. All the above-mentioned qualified signaling pathways are significantly enriched in the high-CASP4-expression phenotype.

GSEA was performed to identify the CASP4-related signaling pathways in the high- and low-CASP4-expression groups, with the nominal *P <*0.05 and the threshold value being a normalized concentration score >1.5 as criteria in the analysis. As shown in **Figure 8** and **Supplementary Table S3**, 14 qualified signaling pathways with significant abundance in high-CASP4-expression phenotypes were identified; among these, 12 pathways were related to immune activation (*e*.*g*., neutrophil degranulation, TOLL, NOD, antigen processing and presentation, natural killer cell-mediated cytotoxicity, cytokine–cytokine receptor interaction, and FCERI-mediated NF-kB activation; **Figures 8A–H**), and the other two pathways were enriched during the cell cycle (*e*.*g*., cell cycle checkpoints and M-phase; **Figures 8I, J**).

## Discussion

Gliomas have a high incidence of intracranial tumors and a poor prognosis. Microglia, also known as intracranial macrophages, constitute the immune microenvironment of intracranial tumors. Pyroptosis is a late-stage model of programmed cell death. Recent studies have shown that pyroptosis can affect the occurrence and growth of tumors by affecting the immune microenvironment of tumors. Research on the role of pyroptosis in gliomas have been rare. This study found that, compared with normal tissues, glioma tissues have a significantly higher CASP4 expression at both the mRNA and protein levels. Further quantitative real-time polymerase chain reaction, western blotting, and immunohistochemical staining confirmed these results. Moreover, Cox regression analysis indicated that CASP4 expression was independently associated with tumor grade, age, histological type, 1p/19q co-deletion, IDH status, and primary treatment outcomes, indicating that it is related to glioma progression. Univariate Cox regression analysis showed that CASP4 may be an independent factor affecting the prognosis of patients with glioma. In addition, ROC analysis provided a high degree of credibility for the diagnostic value of CASP4 expression in patients with gliomas. Collectively, these findings indicated that CASP4 may be a prognostic indicator for gliomas. TCGA analysis of the relationship between CASP4 expression and glioma survival suggested that a high CASP4 expression was related to poor OS, and the same result was observed in gliomas of different grades. Therefore, a high CASP4 expression indicates poor prognosis in glioma patients.

To describe the level of immune infiltration in gliomas, we further evaluated the association between CASP4 expression and immune cell populations. The results showed that CASP4 expression was strongly correlated with the infiltration of immune cells, including macrophages, neutrophils, eosinophils, antibody–drug conjugates, immature dendritic-like cells, T cells, plasmacytoid dendritic cells, Tgd, NK CD56 bright cells, Tcm, TFH, TReg, and CO8+ T cells. The interaction of immune cells changes the immune microenvironment of tumor cells, which affects tumor occurrence and development and therapeutic outcomes (28). The present study demonstrated that CASP4 participated in the regulation of immune infiltration in the local microenvironment of gliomas. To further explore the molecular regulatory mechanism of CASP4 in gliomas, we used TCGA data for the GO and KEGG analysis of the CASP4-coexpressed genes and the GSEA analysis of CASP4. In the GO analysis, BPs related to cell signaling functions were identified, including regulation of neutrophil activation and neutrophil-mediated immunity. Cell composition and molecular function were related to the collagen-containing extracellular matrix, cytokine binding, and cytokine receptor activity. Recent research also suggests that gliomas derived from extracellular matrix-activated microglia secrete IL-18 to enhance the migration of glioma cells (33).

The KEGG analysis revealed that phagosome and EBV infection were the most significantly enriched pathways. EBV infection is considered to be involved in tumorigenesis and development in many tumor types, such as post-transplant lymphoproliferative disorder (34) and gastric cancer (35). EBV has been shown to induce a distinct and immunosuppressive tumor microenvironment to its benefit by influencing and interacting with different components in the tumor microenvironment (36). The differential gene analysis in the current study showed that CASP4 expression is associated with several other important molecules of pyroptosis, including GSDMD, CASP1, IL-18, and TLR2, in gliomas. The GSEA analysis indicated that the genes related to CASP4 expression were mainly enriched in immune cell activation-related and cell cycle-related pathways. This indicated that CASP4 plays an important role in infiltrating immune cells to affect the composition of the tumor immune microenvironment. Overall, the results support that CASP4 participates in glioma prognosis by activating immune cells.

As macrophages of the CNS, microglia play an important role in mediating the immune regulation of the CNS. Based on a previous database search and some experiments, we can conclude that non-classical pyroptosis mediated by CASP4 plays a vital role in the occurrence and development of glioma. It may affect the tumor immune microenvironment by influencing the infiltration of tumor-associated macrophages into the brain and activating the immune response.

**Figure 9:**
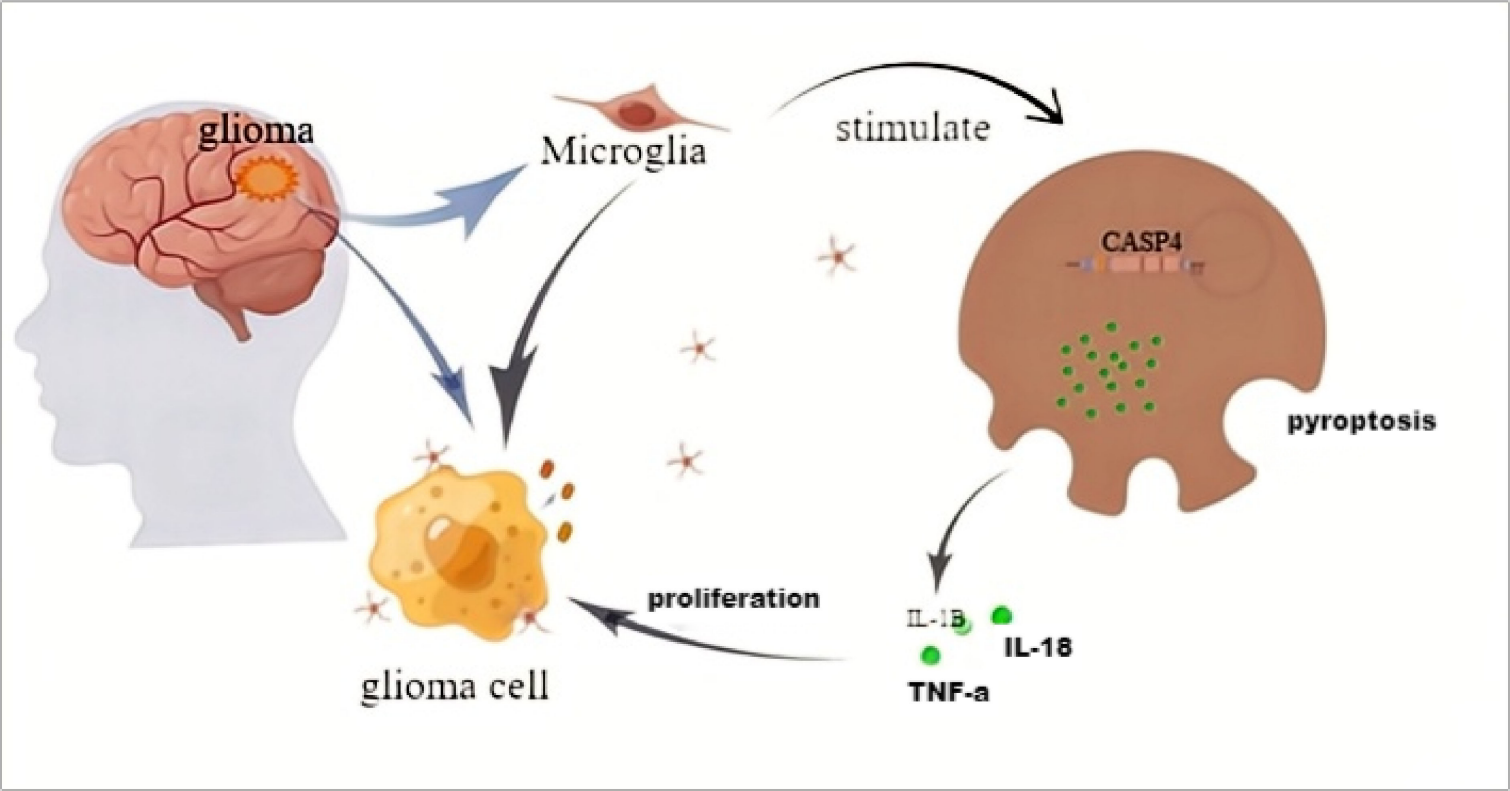
The schematic model of CASP4 affects the distribution of microglia and the proliferation of gliomas.

Glioma tissues contain nearly 30% microglia. When the body is stimulated by certain factors, such as LPS, microglia can be activated and differentiate into a certain phenotype. Activated CASP4 can induce the microglia pyroptosis. The IL-1β, IL-18, TNF-α, and other cytokines in microglia are released and act on glioma to promote tumor cell proliferation. CASP4 in gliomas can promote tumor cells to release irritant factors and enrich microglia in tumor tissues (**Figure 9**).

However, this study has some limitations. We have not directly verified the relationship between non-classical pathway cell pyrogenesis and macrophage microglia in the CNS, and this needs to be studied in the future.

## Conclusion

CASP4-mediated non-classical pyroptosis plays an important role in gliomas. Therefore, CASP4 expression can be used as a diagnostic molecular marker to evaluate patients with glioma.

## Data availability statement

The datasets presented in this study can be found in online repositories. The names of the repository/repositories and accession number(s) can be found in the article/**Supplementary Material**.

## Ethics statement

The studies involving human participants were reviewed and approved by the Ethics Committee of LanZhou University Second Hospital. The patients/participants provided their written informed consent to participate in this study. Written informed consent was obtained from the individual(s) for the publication of any potentially identifiable images or data included in this article.

## Author contributions

YP, GY, QL, and GT conceived and designed the project. GT, QL, LN, YL, XF, SB, and HW performed the experiments. GT, QL, LN, and HW analyzed the data. GT, GY, QL, and HW drew the diagrams. YP, GY, QL, WK, and GT wrote the manuscript. All authors contributed to the article and approved the submitted version.

## Supplementary material

Supplementary Table 1 Clinicopathological characteristics associated with CASP4 expression in glioma patients.

Supplementary Table 2 Correlation between clinicopathological characteristics and overall survival of glioma patients in the univariate or multivariate Cox regression analysis.Values shown in bold are statistically significant (*P* < 0.05).

Supplementary Table 3 Results of the Gene Set Enrichment Analysis.
