# Supplementary material for: CASP4 can be a diagnostic biomarker and correlated with immune infiltrates in gliomas
Source: Front Oncol. 2023 Jan 11;12:1025065. doi: 10.3389/fonc.2022.1025065 (PMC9874090; doi:10.3389/fonc.2022.1025065)
Supplement: Supplementary Table 1 — Clinicopathological characteristics associated with CASP4 expression in glioma patients. [file Table_1.docx]

Supplementary Material

Supplementary Table 1. Clinicopathological characteristics associated with CASP4 expression in glioma patients

| Characteristic | CASP4 expression | | | | | |
| --- | --- | --- | --- | --- | --- | --- |
|  | TCGA | | | CGGA | | |
|  | Low | High | *P* | Low | High | *p* |
| n | 348 | 348 |  | 163 | 162 |  |
| IDH status, n (%) |  |  | **< 0.001** |  |  | **< 0.001** |
| WT | 27 (3.9%) | 219 (31.9%) |  | 148 (45.7%) | 27 (8.3%) |  |
| Mut | 318 (46.4%) | 122 (17.8%) |  | 15 (4.6%) | 134 (41.4%) |  |
| 1p/19q codeletion, n (%) |  |  | **< 0.001** |  |  | **< 0.001** |
| codel | 154 (22.4%) | 17 (2.5%) |  | 53 (16.7%) | 14 (4.4%) |  |
| non-codel | 194 (28.2%) | 324 (47%) |  | 108 (34.1%) | 142 (44.8%) |  |
| Sex, n (%) |  |  | 0.251 |  |  | 0.448 |
| Female | 157 (22.6%) | 141 (20.3%) |  | 65 (20%) | 57 (17.5%) |  |
| Male | 191 (27.4%) | 207 (29.7%) |  | 98 (30.2%) | 105 (32.3%) |  |
| Race, n (%) |  |  | 0.466 |  |  | 1 |
| Asian | 5 (0.7%) | 8 (1.2%) |  | 163(50%) | 162(50%) |  |
| Black or African American | 14 (2%) | 19 (2.8%) |  | NA | NA |  |
| White | 322 (47.1%) | 315 (46.1%) |  | NA | NA |  |
| Age(years), median (IQR) | 39 (32, 51) | 53 (38.75, 63) | **< 0.001** | 38 (34, 45) | 47 (40, 56) | **< 0.001** |

Values shown in bold are statistically significant (*P*< 0.05)

Supplementary Table 2. Correlation between clinicopathological characteristics and overall survival of glioma patients in the univariate or multivariate Cox regression analysis

| Characteristics | Total(N) | Univariate analysis | |  | Multivariate analysis | |
| --- | --- | --- | --- | --- | --- | --- |
|  |  | Hazard ratio (95% CI) | *P* value |  | Hazard ratio (95% CI) | *P* value |
| WHO grade | 634 |  |  |  |  |  |
| G2 | 223 | Control |  |  |  |  |
| G3 | 243 | 2.999 (2.007-4.480) | **<0.001** |  | 1.720 (1.061-2.789) | **0.028** |
| G4 | 168 | 18.615 (12.460-27.812) | **<0.001** |  | 8.497 (2.351-30.711) | **0.001** |
| Primary therapy outcome | 461 |  |  |  |  |  |
| PD | 112 | Control |  |  |  |  |
| SD | 147 | 0.440 (0.294-0.658) | **<0.001** |  | 0.491 (0.295-0.818) | **0.006** |
| PR | 64 | 0.170 (0.074-0.391) | **<0.001** |  | 0.196 (0.069-0.553) | **0.002** |
| CR | 138 | 0.133 (0.064-0.278) | **<0.001** |  | 0.170 (0.078-0.371) | **<0.001** |
| Histological type | 695 |  |  |  |  |  |
| Glioblastoma | 168 | Control |  |  |  |  |
| Oligoastrocytoma | 134 | 0.097 (0.064-0.147) | **<0.001** |  | 2.267 (1.216-4.227) | **0.010** |
| Astrocytoma | 195 | 0.147 (0.107-0.203) | **<0.001** |  | 1.599 (0.921-2.777) | 0.096 |
| Oligodendroglioma | 198 | 0.085 (0.060-0.122) | **<0.001** |  |  |  |
| CASP4 | 695 | 3.243 (2.832-3.712) | **<0.001** |  | 1.534 (0.985-2.390) | 0.059 |
| IDH status | 685 |  |  |  |  |  |
| WT | 246 | Control |  |  |  |  |
| Mut | 439 | 0.117 (0.090-0.152) | **<0.001** |  | 0.423 (0.248-0.721) | **0.002** |
| 1p/19q codeletion | 688 |  |  |  |  |  |
| codel | 170 | Control |  |  |  |  |
| non-codel | 518 | 4.428 (2.885-6.799) | **<0.001** |  | 0.856 (0.446-1.642) | 0.640 |

Supplementary Table 3. Results of GSEA

| ID | ES | NES | p.adjust | FDR |
| --- | --- | --- | --- | --- |
| REACTOME_NEUTROPHIL_DEGRANULATION | 0.577 | 1.722 | 0.014 | 0.011 |
| KEGG_NATURAL_KILLER_CELL_MEDIATED_CYTOTOXICITY | 0.554 | 1.593 | 0.014 | 0.011 |
| KEGG_TOLL_LIKE_RECEPTOR_SIGNALING_PATHWAY | 0.562 | 1.597 | 0.014 | 0.011 |
| KEGG_ANTIGEN_PROCESSING_AND_PRESENTATION | 0.621 | 1.729 | 0.014 | 0.011 |
| KEGG_NOD_LIKE_RECEPTOR_SIGNALING_PATHWAY | 0.606 | 1.643 | 0.014 | 0.011 |
| REACTOME_SIGNALING_BY_INTERLEUKINS | 0.583 | 1.742 | 0.014 | 0.011 |
| WP_IL18_SIGNALING_PATHWAY | 0.528 | 1.560 | 0.014 | 0.011 |
| KEGG_CYTOKINE_CYTOKINE_RECEPTOR_INTERACTION | 0.655 | 1.934 | 0.014 | 0.011 |
| KEGG_JAK_STAT_SIGNALING_PATHWAY | 0.575 | 1.670 | 0.014 | 0.011 |
| REACTOME_FCERI_MEDIATED_NF_KB_ACTIVATION | 0.794 | 2.290 | 0.014 | 0.011 |
| REACTOME_M_PHASE | 0.469 | 1.403 | 0.014 | 0.011 |
| REACTOME_CELL_CYCLE_CHECKPOINTS | 0.529 | 1.568 | 0.014 | 0.011 |
| REACTOME_ANTI_INFLAMMATORY_RESPONSE_FAVOURING_LEISHMANIA_PARASITE_INFECTION | 0.623 | 1.832 | 0.014 | 0.011 |
| REACTOME_BASE_EXCISION_REPAIR | 0.612 | 1.722 | 0.014 | 0.011 |
